# Polygenic risk and incident coronary heart disease in a large multiethnic cohort

**DOI:** 10.1016/j.ajpc.2024.100661

**Published:** 2024-03-28

**Authors:** Carlos Iribarren, Meng Lu, Roberto Elosua, Martha Gulati, Nathan D. Wong, Roger S. Blumenthal, Steven Nissen, Jamal S. Rana

**Affiliations:** a Kaiser Permanente Northern California Division of Research, Oakland, CA, USA; b Cardiovascular Epidemiology and Genetics, Institut Hospital del Mar d'Investigacions Mèdiques (IMIM), Spain and CIBER Cardiovascular Diseases (CIBERCV), Barcelona, Spain; c Faculty of Medicine, University of Vic-Central University of Catalonia (UVic-UCC), Vic, Spain; d Barbra Streisand Women's Heart Center, Smidt Heart Institute, Cedars-Sinai Medical Center, Los Angeles, CA, USA; e Heart Disease Prevention Program, Division of Cardiology, Department of Medicine, University of California Irvine, Irvine, CA, USA; f Ciccarone Center for the Prevention of Cardiovascular Disease, Johns Hopkins School of Medicine, Baltimore, MD, USA; g Cardiovascular Medicine, Cleveland Clinic, Cleveland, OH, USA; h Department of Cardiology, The Permanente Medical Group, Kaiser Permanente Oakland Medical Center, Oakland, CA, USA

**Keywords:** Polygenic risk score, Coronary heart disease, Clinical utility, Primary prevention

## Abstract

**Objective:**

Many studies support the notion that polygenic risk scores (PRS) improve risk prediction for coronary heart disease (CHD) beyond conventional risk factors. However, PRS are not yet considered risk-enhancing factor in guidelines. Our objective was to determine the predictive performance of a commercially available PRS (CARDIO inCode-Score®) compared with the Pooled Cohorts Equations (PCE) in a contemporary, multi-ethnic cohort.

**Methods:**

Participants (*n* = 63,070; 67 % female; 18 % non-European) without prior CHD were followed from 2007 through 12/31/2022. The association between the PRS and incident CHD was assessed using Cox regression adjusting for genetic ancestry and risk factors. Event rates were estimated by categories of PCE and by low/intermediate/high genetic risk within PCE categories; risk discrimination and net reclassification improvement (NRI) were also assessed.

**Results:**

There were 3,289 incident CHD events during 14 years of follow-up. Adjusted hazard ratio (aHR) for incident CHD per 1 SD increase in PRS was 1.18 (95 % CI:1.14–1.22), and the aHR for the upper vs lower quintile of the PRS was 1.66 (95 % CI:1.49–1.86). The association was consistent in both sexes, in European participants compared with all minority groups combined and was strongest in the first 5 years of follow-up. The increase in the C-statistic was 0.004 (0.747 vs. 0.751; *p* < 0.0001); the NRI was 2.4 (0.9–3.8) for the entire cohort and 9.7 (7.5–12.0) for intermediate PCE risk individuals. After incorporating high genetic risk, a further 10 percent of participants at borderline/intermediate PCE risk would be candidates for statin therapy.

**Conclusion:**

Inclusion of polygenic risk improved identification of primary prevention individuals who may benefit from more intensive risk factor modification.

## Graphical abstract

**Figure:**
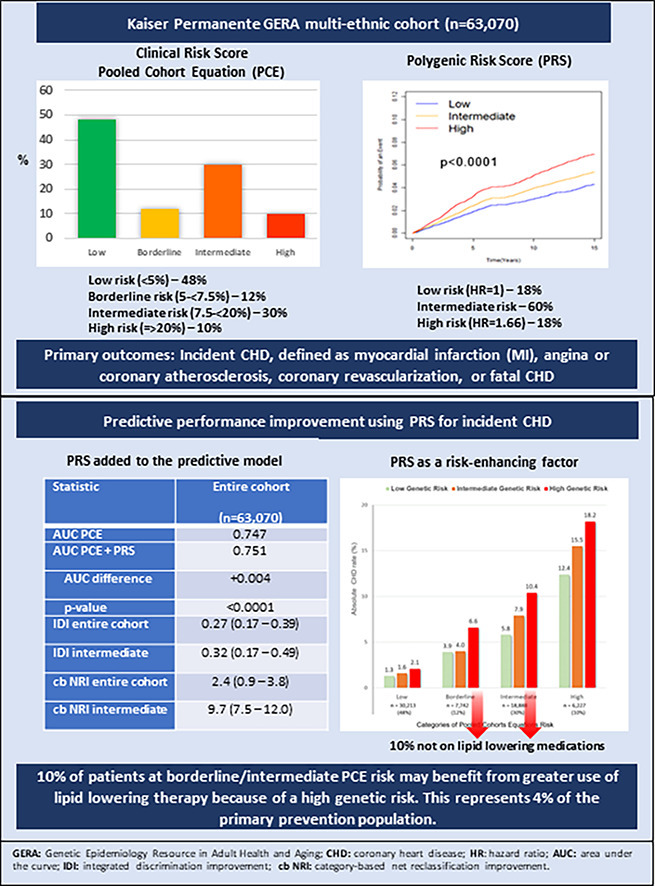

## 1 Introduction

Addressing modifiable risk factors can reduce the incidence of cardiovascular disease (CVD) [1]. However, the prevalence of risky lifestyle behaviors and subsequent cardiometabolic disease is increasing among US young adults [2]. Improving risk assessment and intervention for primordial or primary prevention of CVD remains a challenge. The existing paradigm of clinical care to guide lipid-lowering treatment decisions recommends using the pooled cohort equations (PCEs) to estimate 10-year atherosclerotic cardiovascular disease (ASCVD) risk and is mostly limited to adults 40 to 75 years of age [3].

An increasing body of evidence suggests that polygenic risk scores (PRS) may improve risk prediction for CVD beyond conventional risk factors and may have the advantage of risk assessment at an earlier age [4], [5], [6], [7], [8], [9], [10], [11]. Previously, we reported findings from the Genetic Epidemiology Resource in Adult Health and Aging (GERA) cohort providing evidence that a PRS comprising 12 single nucleotide polymorphisms (SNPs) had comparable predictive performance for incident coronary heart disease (CHD) as compared with PRS based on 36 or 51 SNPs [12,13]. In the current study, we further evaluate longer-term CHD risk associated with this 12-SNP PRS across diverse ethnicities including European, African-American, Latino and Asian using updated follow-up data from the GERA cohort through December 31, 2022.

## 2 Methods

### 2.1 Study cohort

This study made use of genome-wide genetic data obtained from the GERA cohort, comprising 110,266 adult male and female members of Kaiser Permanente of Northern California (KPNC). Detailed information about the cohort has been previously published [14]. The GERA cohort consisted of participants from the larger Research Program on Genes, Environment, and Health (RPGEH) cohort who provided a saliva sample (19 % of the total). The study received approval from the Kaiser Foundation Research Institute Institutional Review Board, and all subjects provided informed consent.

In 2007–8, all RPGEH participants completed a self-administered questionnaire, which included data on medical history, ancestry, health behaviors (smoking, diet, physical activity, and reproductive history), family history of heart attack (in either parent at any age) as well as current weight and height. Out of the 110,266 subjects, 97,973 had complete genetic data necessary for estimating the PRS. Sequential exclusions were applied to this subgroup: 8416 individuals were excluded for ages younger than 30 or older than 74; 2610 individuals were excluded for a prior diagnosis of coronary heart disease (CHD); and 23,877 individuals were excluded for missing data for one or more components of the Pooled Cohorts Equations (PCE). This resulted in a final analytical cohort comprising 63,070 individuals (see consort diagram in **Supplemental Figure 1**).

### 2.2 Outcomes of interest

The primary outcome of interest was the occurrence of incident CHD from the baseline period (2007–09) through December 31, 2022. The primary study endpoint did not include ischemic stroke because the 12-SNP PRS was originally developed to predict CHD and not ischemic stroke [15]. Incident CHD was ascertained using a hospital primary discharge diagnosis of myocardial infarction, angina or coronary atherosclerosis, coronary revascularization procedures (coronary bypass or percutaneous intervention), or death due to CHD. The International Classification of Diseases, Ninth and Tenth Revisions (ICD-9 and ICD-10) codes were used for event ascertainment (Table 1 **of Supplemental Methods)**. The validity of these codes has been demonstrated in prior studies conducted within the KPNC population [16,17]. For angina or coronary atherosclerosis events occurring after 2014 and coded using ICD-10, evidence of significant coronary stenosis >50 % on angiography was required. In the case of myocardial infarction diagnosis, elevated peak troponin levels (>0.40 ng/L) were also required during review of electronic medical records by one of the MD investigators (C.I.)

**Table 1:** Baseline Characteristics of the GERA Cohort (*n* = 63,070).

Characteristics	Mean ± SD or (%)
Age, years (mean ± SD) and n (%)	58.7 ± 9.4
30 – 54	19,869 (31.5 %)
55 – 64	23,600 (37.4 %)
65 – 74	19,601 (31.1 %)
Sex, n (%)	
Male	20,569 (32.6 %)
Female	42,501 (67.4 %)
Self-reported Race/Ethnicity, n (%)	
European	51,839 (82.2 %)
African-American	2084 (3.3 %)
Latino	4347 (6.9 %)
Asian	4800 (7.6 %)
Education level, n (%)	
Less than college	8594 (13.6 %)
College or higher	50,776 (80.5 %)
Missing	3700 (5.9 %)
Smoking status, n (%)	
Never	37,291 (59.1 %)
Former	22,680 (36.0 %)
Current	3099 (4.9 %)
Body mass index, kg/m^2^ (mean ± SD) and n (%)	27.1 ± 5.7
<18	401 (0.6 %)
18–24.9	24,197 (38.4 %)
25–29.9	21,571 (34.2 %)
≥30	14,746 (23.4 %)
Missing	2155 (3.4 %)
Diabetes mellitus, n (%)	8597 (13.6 %)
Hypertension, n (%)	30,678 (48.6 %)
Total Cholesterol, mg/dL (mean ± SD)	197 ± 36
HDL-C, mg/dL (mean ± SD)	56 ± 16
Total cholesterol/HDL ratio (mean ±SD)	3.7 ± 1.05
Cholesterol lowering drugs, n (%)	21,094 (33.4 %)
Pooled Cohort Equations Risk, n (%)	
Low (< 5 %)	30,213 (47.9 %)
Borderline (5 - < 7.5 %)	7742 (12.3 %)
Intermediate (7.5 - <20 %)	18,888 (29.9 %)
High (≥ 20 %)	6227 (9.9 %)
Family history of heart attack, n (%)	
Yes	18,486 (29.3 %)
No	42,866 (68.0 %)
Missing	1718 (2.7 %)

### 2.3 Genotyping and PRS definition

Genotyping was performed at the Institute for Human Genetics, University of California San Francisco, using custom-designed Affymetrix Axiom arrays as previously published [12,13,17,19]. The genome-wide arrays yielded high-quality genotypes, with an average genotype call rate of 99.7 % and SNP reproducibility of 99.9 % [18]. Details regarding SNP selection and the calculation of the polygenic risk score (CARDIO inCode-Score® CHD PRS, GENinCode US Inc.) based on 12 SNPs can be found in Table 2 **and Equation 1 in Supplemental Methods.**

**Table 2:** Association Between a 12-SNP Polygenic Risk Score and Incident CHD Among GERA Subjects.

PRS_12	Number of subjects	Number of events	Age-adjusted rate per 10,000 person-years	Age- and 10 PC of ancestry-adjusted hazard ratio (95 % CI)	Fully-adjusted* hazard ratio (95 % CI)
Per 1 SD	63,070	3289	NA	1.18 (1.14 - 1.22)	1.18 (1.14 - 1.22)
Quintile 1	12,803	529	26.3	1.00	1.00
Quintile 2	12,882	606	30.6	1.14 (1.02 - 1.29)	1.17 (1.04 - 1.31)
Quintile 3	13,530	705	33.5	1.27 (1.13 - 1.42)	1.29 (1.15 - 1.44)
Quintile 4	12,512	687	35.9	1.34 (1.20 - 1.50)	1.38 (1.23 - 1.55)
Quintile 5	11,343	762	43.6	1.65 (1.47 - 1.84)	1.66 (1.49 - 1.86)

PC: principal components.

*age, 10 principal components of genetic ancestry, sex, education level, smoking status, BMI, diabetes, hypertension, TC/HDL ratio, cholesterol lowering drugs.

**Fig. 1:**
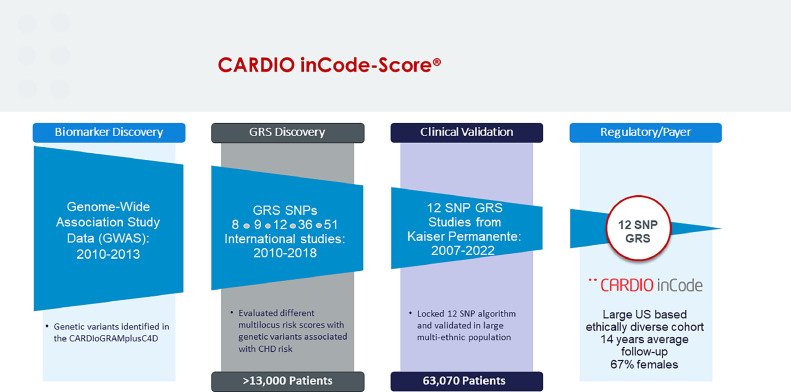
CARDIO inCode-Score® CHD PRS development.

The SNPs included in the CARDIO inCode-Score® CHD PRS were selected through a rigorous process that included multiple studies over more than 10 years [12,13,15,19,20], starting with genome-wide panels of SNPs identified by the CARDIoGRAMplusC4D (Coronary Artery Disease Genome Wide Replication And Meta-Analysis Plus the Coronary Artery Disease [C4D] Genetics) Consortium and eventually settling on a 12 SNP panel (see Fig. 1). Importantly, all 12 SNPs are associated with CHD and are independent of classic risk factors (i.e., low-density lipoprotein cholesterol, high-density lipoprotein cholesterol, blood pressure, smoking and diabetes mellitus), which is not the case for most of the SNPs included in genome-wide PRSs. The CARDIO inCode-Score® CHD PRS has been developed by GENinCode Plc for clinical use in a primary preventive setting and is commercially available in Europe. It has been shown to improve risk reclassification in a multi-ethnic population particularly at intermediate ASCVD risk, when including the PRS in the Framingham risk function [12,13]. In the secondary preventive setting, it has been associated with a higher risk of recurrence in patients with a first myocardial infarction [19,20]. CARDIO inCode-Score® is a ‘first in class’ PRS and has recently been granted *De Novo* status by the FDA and is currently progressing through to regulatory approval.

### 2.4 Pooled cohort equations risk estimation

The 10-year risk of atherosclerotic CVD (non-fatal myocardial infarction, CHD death and ischemic stroke) was determined using the Pooled Cohorts Equations (PCE) [21]. Relevant demographic and clinical information, including age, sex, race/ethnicity, smoking status, body mass index (BMI), and family history of heart disease, were obtained from the RPGEH survey (education level, BMI and family history of heart disease are not used in the PCE). Systolic and diastolic blood pressure measurements were collected from primary care outpatient visits closest to the survey date, and lipid panels (taken closest to the survey date) were acquired from the health plan laboratory database. Diabetes status was determined by cross-referencing with the KPNC diabetes registry [22]. Information regarding hypertension and hypercholesterolemia treatment was ascertained using the Pharmacy Information Management System (PIMS), which relied on prescription dispensing records of drugs belonging to the corresponding therapeutic class, obtained either at the time of the RPGEH survey or up to two years prior.

### 2.5 Statistical analyses

We utilized standard parametric and non-parametric methods to compare the characteristics of cohort members across quintiles of the polygenic risk score (PRS). Subsequently, we used Kaplan-Meier plots depicting the absolute rate of CHD over time in three groups: low genetic risk (quintile 1), intermediate genetic risk (quintiles 2–4), and high genetic risk (quintile 5). Details of the selection of cutoff values are given in Supplemental Methods. Log-rank tests were performed to compare the survival trends across PRS groups. We assessed the association between the PRS (both as a continuous variable in standard deviation units and as quintiles with the lowest quintile as the reference group) and incident CHD using the Cox proportional hazards model [23]. Further analysis of CHD events was censored after the first occurrence of incident CHD, death, or disenrollment from the health plan.

The first model was adjusted for age and 10 principal components of genetic ancestry [24]. The fully-adjusted model included additional covariates for education level, smoking status, BMI, diabetes, hypertension, total cholesterol/high-density lipoprotein cholesterol (TC/HDL-C) ratio, and use of lipid-lowering drugs. To evaluate the proportionality of hazards assumption, we examined Schoenfeld residuals plotted against time and tested the interaction between the PRS and follow-up time. There was visual evidence of downward departure from zero slope at the tail end of follow-up (**Supplemental Figure 2**) and the interaction between PRS and follow-up time was statistically significant (*p* < 0.001), indicating that the PRS–CHD association varied with time. Accordingly, we performed separated modeling considering time up to 5 years, up to 10 years, up to 15 years, and up to 20.6 years.

The analysis was repeated separately in men and women and within each race/ethnic group. We also evaluated the incremental risk stratification based on genetic background by estimating the absolute rates of CHD in PCE groups and, within those groups, based on low/intermediate/high genetic burden. To assess the clinical utility of the PRS as a risk-enhancing factor in the context of the current ASCVD risk assessment standard of care and to compare with findings by Aragam et al., we estimated the percentage of individuals in the PCE borderline /intermediate risk groups that had a PRS in the 5th quintile and were not on statin treatment [4]. To assess the incremental utility of the PRS beyond the PCE, we calculated for the entire cohort, and sex and race/ethnic subgroups, Harrell's C-statistic [25], the integrated discrimination improvement (IDI) index [26], and the category-based (with 4 categories as shown in Table 1) and category-free net reclassification improvement (NRI) [27].

## 3 Results

At baseline, the cohort had a mean (SD) age of 59 [9] years, and 67 % of the participants were female (Table 1). The preponderance of females reflects their greater participation in the RPGEH survey. The majority of the cohort, approximately 82 %, was identified as of European, while 3 % self-identified as African-American, 7 % as Latino, and 8 % as Asian. More than 80 % of the study participants had obtained a college education or higher. About 5 % reported being current smokers, while 36 % were former smokers. The prevalence of diabetes was 14 %, and 23 % had a BMI within the obesity range. Approximately 49 % of the participants had hypertension, and approximately one third were using lipid-lowering agents. In terms of the PCE risk, 48 % of the cohort had a low risk, 12 % had a borderline risk, 30 % had an intermediate risk, and 10 % had a high risk. Additionally, 29 % of the participants reported a family history of heart attack.

The baseline characteristics of the cohort according to quintiles of the PRS are shown in **Supplemental Table 1**. The only notable differences that emerged were greater representation of individuals of European ancestry in quintile 5 (90 %) compared with quintile 1 (79 %), and greater family history of heart attack in quintile 5 (32 %) compared with quintile 1 (27 %). Statistically significant differences were observed for BMI and use of cholesterol lowering drugs across quintiles of PRS. There was no significant association of the PRS with the PCE risk (Chi-square *p* = 0.27).

Box-plots of PRS values by ethnicity are shown in **Supplemental Figure 3.** The median PRS was higher in Europeans, lower in African-Americans, and intermediate in Latinos and Asians (*p* < 0.0001). The distributions of the PRS among subjects who subsequently developed incident CHD (“cases”) and subjects who remained free of CHD (“controls”) are shown in **Supplemental Figure 4**. The PRS was higher in cases compared with controls, a difference (SD; p) of 0.04 (0.21; *p* < 0.001).

**Fig. 2:**
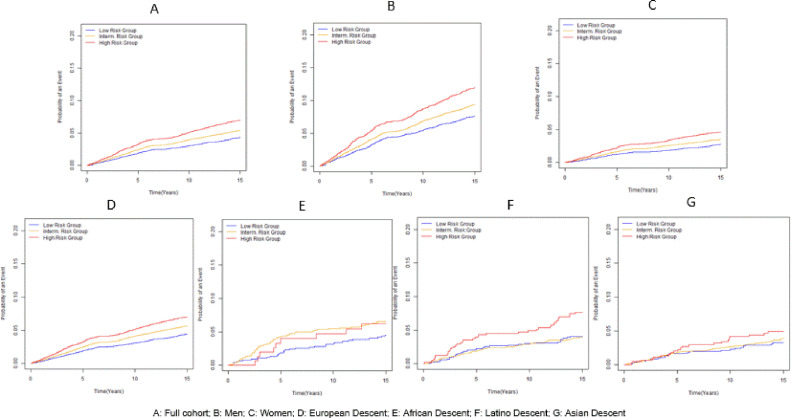
Cumulative absolute risk of CHD over time overall and by sex and race/ethnicity.

After a mean (SD, maximum) follow-up time of 13.8 (3.8, 20.6) years, 3289 incident CHD events were documented. As shown in Fig. 2, there were statistically significant (all *p* < 0.004) separations of absolute rates of CHD over time by genetic risk groups in the entire cohort (panel A), male (panel B), female (panel C), Europeans (panel D), Latinos (panel F) and all minority groups combined (panel H). In Latinos and Asians, because of much smaller sample size, the lines for intermediate and low genetic risk did not diverge from each other, and in African-Americans the high genetic risk group did not diverge from the intermediate risk group. There was a positive linear association between age-adjusted rates of CHD and quintiles of the PRS (Table 2).

In the age-adjusted model, each SD increment in the PRS was associated with 1.18 increased hazard of incident CHD. Adjustment for 10 principal components of genetic ancestry, education level, smoking, BMI, diabetes, hypertension, TC/HDL ratio and use of cholesterol lowering drugs did not materially alter the strength of association. When the PRS was modeled as quintiles, quintile 5 (vs. quintile 1) was associated with hazard ratios (95 % CI) of 1.65 (95 % CI, 1.47–1.84) in the age-adjusted model and with 1.66 (95 % CI, 1.49–1.86) in the fully-adjusted model.

In analyses stratifying by sex (**Supplemental Table 2a**), the aHRs (95 % CI) were 1.64 (95 % CI, 1.42 - 1.90) in males and 1.69 (95 % CI, 1.42 - 2.00) in females.

In analysis stratifying by self-reported ethnicity (**Supplemental Table 2b)**, the aHRs were 1.66 (95 % CI, 1.47–1.88) in Europeans, 1.50 (95 % CI, 0.71–3.14) in African Americans, 2.07 (95 % CI, 1.32–3.24) in Latinos and 1.36 (95 % CI, 0.78–2.37) in Asians. For all minority groups combined, the aHR (95 % CI) was 1.68 (95 % CI, 1.24–2.29). When the analysis was performed segmenting follow-up time into 5-year increments, we observed a stronger association of the PRS with incident CHD during the first 5 years (Q5 vs Q1 aHR=1.91; 95 % CI, 1.63–2.24) which slightly diminished in strength when considering up to 10 years (Q5 vs Q1 aHR=1.81; 95 % CI, 1.59–2.06), up to 15 years (Q5 vs Q1 aHR=1.70; 95 % CI, 1.51–1.90) and the entire follow- up to 20.6 years (Q5 vs Q1 aHR=1.66; 95 % CI, 1.49–1.86) (**Supplemental Table 2c**).

**Fig. 3:**
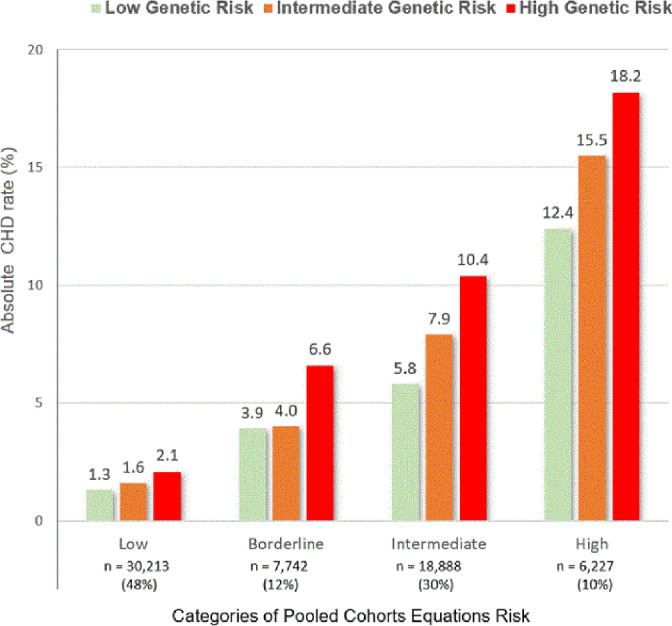
Absolute CHD risk (%) by joint categories of PCE risk and polygenic risk.

Fig. 3 shows the absolute CHD rate according for each category of PCE risk and genetic status. Within each PCE group there was incremental risk stratification by genetic risk, more evident in borderline and intermediate risk categories. Of note, those at borderline PCE risk with high genetic risk had a higher event rate than those at intermediate PCE risk but low genetic risk (6.6 vs. 5.8 %). In addition, event rates for those at intermediate PCE risk with high genetic risk approximated the rate of those at high PCE risk but low genetic risk (10.4 vs. 12.4 %).

To assess the potential additive value of CHD genetic risk status, we segregated those in quintile 5 of the PRS (the ‘high genetic risk group’) into PCE groups. Within these PCE categories, we assessed whether these individuals were receiving stain treatment or not (Table 3) to determine whether borderline/intermediate risk individuals might benefit from statin treatment on the basis of a high genetic risk. In the entire cohort, 42.2 % of individuals were at borderline/intermediate PCE risk and this proportion was very similar (42.3 %) among those classified as being in the high genetic risk group. Among borderline/intermediate PCE risk subjects with high genetic risk (*n* = 4799), 2658 of 26,630 (10 %) were not taking statins. Corresponding proportions were 10.7 % (2436/22,649) in the European, 4.2 % (45/1075) in the African-American, 7.7 % (107/1383) in the Latino and 4.8 % (70/1523) in the Asian cohorts, respectively (**Supplemental Table 3**).

**Table 3:** PCE Risk Groups in the High Polygenic Risk Group (Quintile 5 of the PRS) by Statin Use in the GERA Cohort (*n* = 63,070).

	Quintile 5 of the PRS			
PCE Risk Groups	Statin Use		All	Entire Cohort
	No	Yes		
Low (< 5 %)	4420 (59.3 %)	1021 (26.2 %)	5441 (48.0 %)	30,213 (47.9 %)
Borderline (5 to < 7.5 %)	832 (11.2 %)	517 (13.3 %)	1349 (11.9 %)	7742 (12.3 %)
Intermediate (7.5 to < 20 %)	1826 (24.5 %)	1624 (41.7 %)	3450 (30.4 %)	18,888 (29.9 %)
High (>= 20 %)	371 (5.0 %)	732 (18.8 %)	1103 (9.7 %)	6227 (9.9 %)
All	7449 (100.0 %)	3894 (100.0 %)	11,343 (100.0 %)	63,070 (100.0 %)

In the entire cohort, the Harrell C statistic after the addition of the PRS to a model already containing the PCE increased only marginally (0.747 vs. 0.751; difference in C statistic=+0.004; *p* < 0.0001). The IDI was 0.27 (0.17–0.39) in the entire cohort and 0.32 (0.17–0.49) in individuals at intermediate PCE risk. The category-based NRI was 2.4 (0.9–3.8) in the entire cohort and 9.7 (7.5–12.0) in individuals at intermediate PCE risk. The category-free NRI was 16.2 (12.7–19.9) in the entire cohort and 16.7 (12.0–21.8) in intermediate PCE risk individuals. The corresponding results in sex and race/ethnic subgroups are provided in **Supplemental Table 4**. Results were similar in males and females. However, reclassification parameters were lower and less precise in minority groups.

## 4 Discussion

The PRS used in the current study evaluated 12 SNPs (CARDIO inCode-Score®) in a wide range of racial and ethnic groups and uses the PCE rather than the Framingham risk score used in prior studies [12,13]. This PRS was independently associated with CHD incidence, with an adjusted hazard ratio of 1.66 for the highest quintile relative to the lowest quintile. This association was consistent between sexes and race/ethnic groups (European and all minorities combined). However, we observed some heterogeneity across the ethnic groups, with a weaker association in Asians and African-Americans and a stronger association in Latinos.

The PRS–CHD association appeared to be time-dependent with the strongest predictive value in the first 5 and 10 years compared to longer follow-up at 15 or 20 years. These findings suggest that the genetic influence on risk of CHD may be more evident in the shorter term compared with the distant future, perhaps related to the role of genetic determinants in premature CHD. Consistent with this argument, the age at CHD presentation tended to be younger with shorter follow-up time (**Supplemental Table 2 (c)**). This is also consistent with the findings of another study that suggested that the predictive ability of a PRS was greater in younger individuals and optimally used to identify patients with borderline and intermediate clinical risk who should initiate statin therapy and intensify their lifestyle modification efforts [9].

In the current analyses, the PRS derived genetic risk provided additional risk stratification within PCE risk categories. Individuals with intermediate risk by PCE (7.5 to < 20 %) but with high genetic risk had an absolute risk similar to those with high PCE risk but low genetic risk. Accordingly, these results show potentially useful reclassification with an IDI of 0.32 and a category-based NRI of 9.7 in the intermediate PCE group. We observed lower reclassification indices in minority groups, particularly African-Americans, which may be attributable to much smaller sample sizes. The current data indicates that an additional 10 % of patients at borderline/intermediate PCE risk may benefit from greater use of statin therapy and more aggressive lifestyle modifications because of a high genetic risk. The increase in the AUC after adding the PRS to a Cox model containing the PCE was modest (0.004) but statistically significant.

The PCE is applicable to ages 40 and above and based on presence or absence of clinical risk factors at any specific time point. By contrast, the PRS provides incremental predictive insights in younger patients, does not change over time, and therefore provides a lifetime risk assessment.

Our results are in general agreement with several major studies published in the last 5 years that have analyzed the association between various PRS's and CHD endpoints [[4], [5], [6], [7], [8], [9], [10], [11],^28^]. Six of these studies relied on data from the UK Biobank [5,6,[8], [9], [10], [11]], which is a predominantly European cohort. The number of SNPs varied from 241 in the Marston et al. study [9] to over 6.6 million in the Khera et al. [8] study. In these UK Biobank studies, the reported adjusted standardized HRs were higher than ours (1.18) and ranged between 1.71 in the Inouye et al. study [6] and 1.73 in the Patel et al. study [10]. The lower predictive value of our PRS may reflect the lower number of SNPs. Elliot et al. [5]. and Riveros-McKay et al. [11]. studies reported overall net reclassification improvements of 4 and 5.9, respectively, which is higher than overall net reclassification in the current study (2.4). Marston et al. [9] reported a net reclassification improvement index for patients of borderline to intermediate risk of 0.16 (95 % CI, 0.15–0.17) [9]. Wang et al. validated a South-Asian-specific 6.6 million DNA variants PRS in the BRAVE (Bangladesh Risk of Acute Vascular Events) study [28]. In this study, the OR per SD of the PRS was 1.60 (*p* < 0.001), but it was only adjusted for age, sex and genetic ancestry. Of note, none of these studies reported the NRI in the intermediate PCE risk group (9.7 in our cohort), which is the subgroup in whom risk enhancing factors are recommended to inform treatment decisions.

Only 2 studies were based on US populations, the Aragam et al. study [4] across 3 health care systems and the Khan et al. study [7] based on the MESA and Rotterdam cohorts. The Aragam et al. study concluded that an additional 4.1 % of primary prevention patients (and 11 % of those in PCE borderline/intermediate risk groups) may be recommended for statin therapy if high CAD PRS were considered a risk-enhancing factor. Corresponding estimates in our GERA cohort are similar: 4.2 % of the primary prevention population and 10 % of the borderline/intermediate PCE risk individuals. The Khan et al. study concluded that coronary artery calcium (CAC) screening had much better predictive performance and reclassification properties than a 6.6 million SNPs PRS in the MESA cohort with a mean age of 60. However, the comparison of CAC and PRS for CAD prediction is applicable only when coronary calcium has potentially developed (typically over age 45). CAC score signifies late-stage calcified coronary plaque, whereas PRS does not change over time, provides risk assessment from birth, and can be applied for younger adults before CAC is present. Other considerations are access to CT scanning and modest radiation exposure.

The ACC/AHA Guideline on the Primary Prevention of CVD advises consideration of a statin treatment to lower LDL-C by 50 % in high-risk individuals (i.e., 10-year ASCVD risk ≥ 20 %), and initiating a risk discussion in individuals with borderline (10-year ASCVD risk 5 - < 7.5 %) and intermediate (10-year ASCVD risk 7.5 – < 20 %) risk, favoring moderate-intensity statin treatment if risk enhancers are present [29]. This risk assessment is based on clinical and risk enhancing factors that include family history of premature ASCVD. However, our findings suggest that incorporating the PRS may enhance prediction of risk. The PCE is limited to 10-year risk estimation and its applicable for ages 40 and above. A recent study has shown a substantial gradient in risk of future CAC and CHD events according to PRS for CHD assessed in young adulthood, and provided evidence for utility of PRS among individuals with no detectable CAC in middle age [30].

A recent scientific statement from the AHA concludes that the CAD PRS can provide additional prognostic information that may have utility in guiding pharmacological management, particularly for LDL-C lowering [31]. Moreover, CAD PRSs can identify younger individuals who may benefit the most from more aggressive lifestyle modification [32,33]. Accordingly, knowledge of the PRS can motivate individuals to make extensive lifestyle changes, similar to that seen in CAC scoring [34]. A recent study suggest that using CAD-PRS as a risk-enhancing factor may reduce the mean cost per individual, improve quality-adjusted life-years, and potentially avert future events of CAD and ischemic stroke when compared with PCE alone [35].

Our study has several strengths, including the availability of a large, U.S. based ethnically diverse cohort, with more than half females, followed for an average of 14 years. Rather than a health maintenance organization (HMO, claims data), KPNC is an integrated health care delivery system where utilization comes from the systems own hospitals, outpatient clinics, central laboratory and pharmacies. As long as members remain in the plan, ascertainment of inpatient services is essentially complete. Among persons in the GERA cohort, over 97 % have at least 5 years of continuous membership, and over 83 % have at least 10 years of continuous membership with an average duration of membership of 23.5 years [14]. Compared to previous studies, we believe our findings are unique because of the practicality of a 12-SNP PRS that can be undertaken on saliva or blood and its commercial availability. This 12-SNP PRS test is manageable in daily clinical practice, both from the laboratory and the prescriber point of view, as it is a relatively simple technology with a short turnaround time and is commercially available.

We recognize some limitations in our study. First, our cohort participants were all members of KPNC, therefore findings may not fully generalize to uninsured or other populations. Moreover, the GERA cohort has a high representation of the upper end of the educational spectrum, which could limit its generalizability to populations with lower educational levels. Second, no attempt was made to recalibrate the PCE since it may overestimate risk in contemporary populations or samples of non-European ethnicities [36,37]. Third, the 12-SNP PRS was developed and validated using European-based genetic panels and thus is not fully optimized for African-American or Asian subjects, which may explain its reduced predictive performance in these groups in our study. Fourth, we did not have measures of Lp(a), so we were unable to examine the additional contribution of this biomarker to polygenic risk prediction of coronary artery disease as recently shown [38].

In summary, this PRS provided additional predictive performance and improved risk reclassification for incident CHD. Our results support consideration of polygenic risk in predictive algorithms for primary prevention to encourage optimization of lifestyle and provide more precision-based therapy recommendations. Moreover, our results support strong consideration for high polygenic risk to be designated as a risk enhancing factor.

This study was funded by a grant to Dr. Iribarren by GENinCode, Pls.

## CRediT authorship contribution statement

**Carlos Iribarren:** Writing – original draft, Visualization, Validation, Supervision, Resources, Project administration, Methodology, Investigation, Funding acquisition, Data curation, Conceptualization. **Meng Lu:** Writing – review & editing, Visualization, Validation, Methodology, Formal analysis, Data curation. **Roberto Elosua:** Writing – review & editing, Conceptualization. **Martha Gulati:** Writing – review & editing. **Nathan D. Wong:** Writing – review & editing. **Roger S. Blumenthal:** Writing – review & editing. **Steven Nissen:** Writing – review & editing. **Jamal S. Rana:** Writing – review & editing.

## Declaration of competing interest

The authors declare the following financial interests/personal relationships which may be considered as potential competing interests:

Carlos Iribarren reports financial support was provided by GENinCode, Plc. Roberto Elosua is a member of the scientific advisory board of GENinCode, Plc, and inventor in a patent application based on the CARDIOinCODE-Score® CHD PRS whose applicant is GENinCode, Plc. If there are other authors, they declare that they have no known competing financial interests or personal relationships that could have appeared to influence the work reported in this paper.

## Appendix Supplementary materials

Image, application 1

Image, application 2
