# Supplementary material for: Polygenic risk and incident coronary heart disease in a large multiethnic cohort
Source: Am J Prev Cardiol. 2024 Mar 28;18:100661. doi: 10.1016/j.ajpc.2024.100661 (PMC11004687; doi:10.1016/j.ajpc.2024.100661)
Supplement: Supplementary file 1 [file mmc1.docx]

| Condition | Primary Hospital Discharge Diagnosis  ICD-9 Codes | ICD-9 Procedure Codes | CPT4 Codes | Primary Hospital Discharge Diagnosis Codes or Underlying Cause of Death  ICD-10 Codes | IC D-10 Procedure Codes |
| --- | --- | --- | --- | --- | --- |
| Unstable and stable angina pectoris | 411.1, 413.0, 413.1, 413.9 |  |  | I20.0, I24.0, I25.110, I25.119, I25.81, I25.7x |  |
| Acute myocardial infarction | 410.x |  |  | I21.x |  |
| Coronary revascularization procedure |  | 36.01, 36.02, 36.05, 36.06, 36.07, 36.09  36.10, 36.11, 36.12, 36.13, 36.14, 36.15, 36.16, 36.17, 36.19, 36.03 | 33510, 33511, 33512, 33513, 33514, 33515, 33516, 33517, 33518, 33519, 33521, 33522, 33523, 33530, 33533, 33534, 33535, 33536,  92980, 92981, 92982, 92984, 92995, 92996 |  | 021xxxx |
| Coronary heart disease death |  |  |  | I20-I22, I25 |  |

**Supplemental Methods.**

**Table 1.** Codes for Identification of CHD Events.

**Table 2.** Selected genetic variants and their risk alleles

| **Gene** | **Genetic variant** | **Risk allele** | **Source** |
| --- | --- | --- | --- |
| ALOX5AP | rs10507391 | A | Girelli et al, 2007^1^  Linsel-Nitschze et al, 2008^2^ |
|  | rs17222842 | G |  |
|  | rs9315050 | A |  |
|  | rs17216473 | A |  |
| CDKN2A/B | rs1333049 | C | NHGRI GWAS catalog^3^ |
| SLC5A3/KCNE2 | rs9982601 | T | NHGRI GWAS catalog^3^ |
| LPA | rs10455872 | G | Shiffman et al, 2010^4^ |
| TAF1A | rs17464857 | T | NHGRI GWAS catalog^3^ |
| MRAS | rs9818870 | T | NHGRI GWAS catalog^3^ |
| PHACTR1 | rs12526453 | C | NHGRI GWAS catalog^3^ |
| WDR12 | rs6725887 | C | NHGRI GWAS catalog^3^ |
| CXCL12 | rs501120 | T | NHGRI GWAS catalog^3^ |

**Estimated effect size for each genetic variant**

The estimated effect size (weight) for each variant was extracted from the CARDIoGRAMplusC4D Consortium for the 8 individual genetic variants^5^ and from literature-based evidence for the ALOX5AP haplotype B (4 genetic variants analyzed together)^1,2^ (see **Table 2**).

**Estimation of the individual Polygenic Risk Score (PRS)**

The estimation of the individual PRS is computed as the sum of the number of risk alleles across the set of selected genetic variants after weighting each one by the magnitude of its estimated effect size on the phenotype (Equation 1). Each individual might have 0, 1 or 2 risk alleles in each of the variants.

**Equation 1:** Estimation of the individual PRS

$$PRS= \sum_{i=1}^{n} B_{i}\times{SNP}_{i}$$

where:

n = number of SNPs

B*_i_* = estimated effect size reported for each variant

SNP*_i_* = number of copies of the risk allele in each individual SNP evaluated (with values 0, 1, or 2).

The individual PRS values are classified in quintiles, being considered quintile 1 the reference or low genetic risk group, quintile 2 through quintile 4 the intermediate genetic risk group, and quintile 5 the high genetic risk group.

The PRS cut-offs for the different genetic risk groups (quintiles) were defined through the analysis of a simulated population of half a million European non-Finnish individuals from gnomAD^6^.

**References:**

1. Girelli D, Martinelli N, Trabetti E, et al. ALOX5AP gene variants and risk of coronary artery disease: an angiography-based study. Eur J Hum Genet 2007;15(9):959-66.

2. Linsel-Nitschke P, Gotz A, Medack A, et al. Genetic variation in the arachidonate 5-lipoxygenase-activating protein (ALOX5AP) is associated with myocardial infarction in the German population. Clin Sci (Lond) 2008;115(10):309-15.

3. Hindorff LA JH, Mehta JP, Manolio TA. A catalog of published genome-wide association studies: 2009. (<http://www.genome.gov/26525384>).

4. Shiffman D, Louie JZ, Rowland CM, Malloy MJ, Kane JP, Devlin JJ. Single variants can explain the association between coronary heart disease and haplotypes in the apolipoprotein(a) locus. Atherosclerosis 2010;212(1):193-6.

5. Deloukas P, Kanoni S, Willenborg C, et al. Large-scale association analysis identifies new risk loci for coronary artery disease. Nat Genet 2013;45(1):25-33.

6. Karczewski KJ, Francioli LC, Tiao G, et al. The mutational constraint spectrum quantified from variation in 141,456 humans. Nature 2020;581(7809):434-443.
