# Supplementary material for: Polygenic risk and incident coronary heart disease in a large multiethnic cohort
Source: Am J Prev Cardiol. 2024 Mar 28;18:100661. doi: 10.1016/j.ajpc.2024.100661 (PMC11004687; doi:10.1016/j.ajpc.2024.100661)
Supplement: Supplementary file 2 [file mmc2.docx]

**Supplemental Table 1.** Baseline Characteristics of the GERA Cohort (n=63,070) According to Quintiles of the PRS.

|  | Quintile 1 | Quintile 2 | Quintile 3 | Quintile 4 | Quintile 5 | p-value |
| --- | --- | --- | --- | --- | --- | --- |
| N (%) | 12803 (20.3%) | 12882 (20.4%) | 13530 (21.4%) | 12512 (19.8%) | 11343 (18.0%) |  |
| Number of CHD events | 529 (4.1%) | 606 (4.7%) | 705 (5.2%) | 687 (5.5%) | 762 (6.7%) | <.0001^1^ |
| Age (years), mean (SD) | 58.7 (9.52) | 58.6 (9.44) | 58.8 (9.39) | 58.6 (9.38) | 58.9 (9.35) | 0.13^1^ |
| 30-54 | 4074 (31.8%) | 4125 (32.0%) | 4225 (31.2%) | 3957 (31.6%) | 3488 (30.8%) | 0.04^2^ |
| 55-64 | 4671 (36.5%) | 4822 (37.4%) | 5060 (37.4%) | 4777 (38.2%) | 4270 (37.6%) |  |
| 65-74 | 4058 (31.7%) | 3935 (30.5%) | 4245 (31.4%) | 3778 (30.2%) | 3585 (31.6%) |  |
| Sex, n (%) |  |  |  |  |  | 0.93^2^ |
| Male | 4164 (32.5%) | 4186 (32.5%) | 4448 (32.9%) | 4093 (32.7%) | 3678 (32.4%) |  |
| Female | 8639 (67.5%) | 8696 (67.5%) | 9082 (67.1%) | 8419 (67.3%) | 7665 (67.6%) |  |
| Race/Ethnicity, n (%) |  |  |  |  |  | <.0001^2^ |
| European | 10060 (78.6%) | 10360 (80.4%) | 10952 (80.9%) | 10298 (82.3%) | 10169 (89.7%) |  |
| African-American | 775 (6.1%) | 484 (3.8%) | 415 (3.1%) | 250 (2.0%) | 160 (1.4%) |  |
| Latino | 1024 (8.0%) | 996 (7.7%) | 950 (7.0%) | 821 (6.6%) | 556 (4.9%) |  |
| Asian | 944 (7.4%) | 1042 (8.1%) | 1213 (9.0%) | 1143 (9.1%) | 458 (4.0%) |  |
| Education level, n (%) |  |  |  |  |  | 0.32^2^ |
| Less than college | 1788 (14.0%) | 1756 (13.6%) | 1827 (13.5%) | 1713 (13.7%) | 1510 (13.3%) |  |
| College or higher | 10241 (80.0%) | 10378 (80.6%) | 10863 (80.3%) | 10100 (80.7%) | 9194 (81.1%) |  |
| Missing | 774 (6.0%) | 748 (5.8%) | 840 (6.2%) | 699 (5.6%) | 639 (5.6%) |  |
| Smoking status, n (%) |  |  |  |  |  | 0.07^2^ |
| Never | 7581 (59.2%) | 7547 (58.6%) | 8034 (59.4%) | 7502 (60.0%) | 6627 (58.4%) |  |
| Former | 4610 (36.0%) | 4641 (36.0%) | 4848 (35.8%) | 4412 (35.3%) | 4169 (36.8%) |  |
| Current | 612 (4.8%) | 694 (5.4%) | 648 (4.8%) | 598 (4.8%) | 547 (4.8%) |  |
| Body mass index (kg/m^2^), n (%) | 27.3 (5.77) | 27.1 (5.56) | 27.0 (5.64) | 27.1 (5.63) | 27.3 (5.81) | 0.0005^1^ |
| <18 | 80 (0.6%) | 77 (0.6%) | 91 (0.7%) | 66 (0.5%) | 87 (0.8%) |  |
| 18-24.9 | 4723 (36.9%) | 4998 (38.8%) | 5274 (39.0%) | 4880 (39.0%) | 4322 (38.1%) |  |
| 25-29.9 | 4478 (35.0%) | 4384 (34.0%) | 4620 (34.1%) | 4297 (34.3%) | 3792 (33.4%) |  |
| >=30 | 3072 (24.0%) | 2956 (22.9%) | 3119 (23.1%) | 2843 (22.7%) | 2756 (24.3%) |  |
| Missing | 450 (3.5%) | 467 (3.6%) | 426 (3.1%) | 426 (3.4%) | 386 (3.4%) |  |
| Diabetes mellitus, n (%) | 1770 (13.8%) | 1761 (13.7%) | 1899 (14.0%) | 1643 (13.1%) | 1524 (13.4%) | 0.25^1^ |
| Hypertension, n (%) | 6278 (49.0%) | 6204 (48.2%) | 6565 (48.5%) | 6042 (48.3%) | 5589 (49.3%) | 0.34^1^ |
| Total Cholesterol/HDL ratio | 197.1 (36.83) | 197.3 (36.53) | 197.4 (36.48) | 197.6 (36.46) | 198.0 (36.45) | 0.24^2^ |
| HDL-C, mg/dL, Mean (SD) | 56.4 (15.63) | 56.5 (15.88) | 56.4 (15.55) | 56.3 (15.51) | 56.6 (15.80) | 0.87^2^ |
| Total cholesterol/HDL ratio, Mean (SD) | 3.7 (1.05) | 3.7 (1.06) | 3.7 (1.05) | 3.7 (1.07) | 3.7 (1.05) | 0.71^2^ |
| Cholesterol lowering drugs, n (%) | 4148 (32.4%) | 4223 (32.8%) | 4622 (34.2%) | 4207 (33.6%) | 3894 (34.3%) | 0.003^1^ |
| Pooled Cohorts Equation, mean (SD) |  |  |  |  |  | 0.27^1^ |
| Low (< 5%) | 6092 (47.6%) | 6176 (47.9%) | 6425 (47.5%) | 6079 (48.6%) | 5441 (48.0%) |  |
| Borderline (5 - <7.5%) | 1547 (12.1%) | 1570 (12.2%) | 1697 (12.5%) | 1579 (12.6%) | 1349 (11.9%) |  |
| Intermediate (7.5 - <20%) | 3834 (29.9%) | 3888 (30.2%) | 4066 (30.1%) | 3650 (29.2%) | 3450 (30.4%) |  |
| High (≥ 20%) | 1330 (10.4%) | 1248 (9.7%) | 1342 (9.9%) | 1204 (9.6%) | 1103 (9.7%) |  |
| Family history of angina/heart attack |  |  |  |  |  | <.0001^1^ |
| Yes | 3488 (27.2%) | 3622 (28.1%) | 3932 (29.1%) | 3777 (30.2%) | 3667 (32.3%) |  |
| No | 8983 (70.2%) | 8935 (69.4%) | 9239 (68.3%) | 8396 (67.1%) | 7313 (64.5%) |  |
| Missing | 332 (2.6%) | 325 (2.5%) | 359 (2.7%) | 339 (2.7%) | 363 (3.2%) |  |

^1^Chi-Square p-value; ^2^Kruskal-Wallis p-value

**Supplemental Table 2(a).** Association Between Polygenic Risk Score and Incident CHD Among GERA Subjects by Sex.

| PRS_12 | Number of subjects | Number of events | Age-adjusted rate per 10,000 person-years | Age- and 10 PC of genetic ancestry-adjusted hazard ratio  (95% CI) | Fully-adjusted* hazard ratio  (95% CI) |
| --- | --- | --- | --- | --- | --- |
| Male | | | | | |
| Per 1 SD | 20569 | 1850 | NA | 1.17 (1.12 - 1.23) | 1.18 (1.13 - 1.24) |
| Quintile 1 | 4164 | 305 | 48.2 | 1.00 | 1.00 |
| Quintiles 2-4 | 12727 | 1121 | 59.4 | 1.21 (1.07 - 1.38) | 1.25 (1.10 - 1.42) |
| Quintile 5 | 3678 | 424 | 78.8 | 1.60 (1.38 - 1.85) | 1.64 (1.42 - 1.90) |
| Female | | | | | |
| Per 1 SD | 42501 | 1439 | NA | 1.19 (1.13 - 1.25) | 1.18 (1.13 - 1.25) |
| Quintile 1 | 8639 | 224 | 16.0 | 1.00 | 1.00 |
| Quintiles 2-4 | 26197 | 877 | 21.0 | 1.29 (1.12 - 1.50) | 1.31 (1.13 - 1.52) |
| Quintile 5 | 7665 | 338 | 27.4 | 1.72 (1.45 - 2.03) | 1.69 (1.42 - 2.00) |

PC: principal components

*age, 10 principal components of genetic ancestry, education level, smoking status, BMI, diabetes, hypertension, TC/HDL ratio, cholesterol lowering drugs.

**Supplemental Table 2(b).** Association Between a 12-SNP Polygenic Risk Score and Incident CHD Among GERA Subjects by Self-reported Race/Ethnicity.

| PRS_12 | Number of subjects | Number of events | Age-adjusted rate per 10,000 person-years | Age-adjusted hazard ratio  (95% CI) | Fully-adjusted* hazard ratio  (95% CI) |
| --- | --- | --- | --- | --- | --- |
| European | | | | | |
| Per 1 SD | 51839 | 2826 | NA | 1.18 (1.14 - 1.22) | 1.18 (1.14 - 1.23) |
| Quintile 1 | 10060 | 428 | 25.7 | 1.00 | 1.00 |
| Quintiles 2-4 | 31610 | 1705 | 33.4 | 1.28 (1.15 - 1.42) | 1.30 (1.17 - 1.44) |
| Quintile 5 | 10169 | 693 | 43.0 | 1.62 (1.44 - 1.83) | 1.66 (1.47 - 1.88) |
| African-American | | | | | |
| Per 1 SD | 2084 | 112 | NA | 1.09 (0.89 - 1.34) | 1.11 (0.90 - 1.37) |
| Quintile 1 | 775 | 32 | 30.1 | 1.00 | 1.00 |
| Quintiles 2-4 | 1149 | 71 | 47.6 | 1.53 (1.01 - 2.33) | 1.57 (1.03 - 2.39) |
| Quintile 5 | 160 | 9 | 43.7 | 1.40 (0.67 - 2.93) | 1.50 (0.71 - 3.14) |
| Latino | | | | | |
| Per 1 SD | 4347 | 177 | NA | 1.22 (1.05 - 1.42) | 1.24 (1.06 - 1.45) |
| Quintile 1 | 1024 | 38 | 29.6 | 1.00 | 1.00 |
| Quintiles 2-4 | 2767 | 100 | 28.7 | 0.97 (0.67 - 1.41) | 1.02 (0.70 - 1.48) |
| Quintile 5 | 556 | 39 | 56.6 | 1.92 (1.23 - 3.00) | 2.07 (1.32 - 3.24) |
| Asian | | | | | |
| Per 1 SD | 4800 | 174 | NA | 1.13 (0.95 - 1.35) | 1.13 (0.95 - 1.35) |
| Quintile 1 | 944 | 31 | 25.8 | 1.00 | 1.00 |
| Quintiles 2-4 | 3398 | 122 | 28.7 | 1.11 (0.75 - 1.64) | 1.13 (0.76 - 1.68) |
| Quintile 5 | 458 | 21 | 36.0 | 1.42 (0.82 - 2.47) | 1.36 (0.78 - 2.37) |
| All Minority Groups Combined | | | | | |
| Per 1 SD | 11231 | 463 | NA | 1.13 (1.02 - 1.25) | 1.15 (1.04 - 1.27) |
| Quintile 1 | 2743 | 101 | 28.5 | 1.00 | 1.00 |
| Quintiles 2-4 | 7314 | 293 | 31.8 | 1.09 (0.87 - 1.37) | 1.15 (0.92 - 1.44) |
| Quintile 5 | 1174 | 69 | 46.8 | 1.62 (1.19 - 2.20) | 1.68 (1.24 - 2.29) |

*age, sex, education level, smoking status, BMI, diabetes, hypertension, TC/HDL ratio, cholesterol lowering drugs.

**Supplemental Table 2 (c).** Association Between a 12-SNP Polygenic Risk Score and Incident CHD Among GERA Subjects by

Follow-up Time.

| Follow-up time, years | Number of subjects | Number of events | Age at event, Mean (SD) | Adjusted* HR (95% CI)  per 1 SD | Adjusted* HR (95% CI)  Q5 vs. Q1 |
| --- | --- | --- | --- | --- | --- |
| Up to 5 | 63,070 | 1,640 | 67.0 (7.2) | 1.22 (1.16 - 1.27) | 1.91 (1.63 - 2.24) |
| Up to 10 | 63,070 | 2,501 | 68.2 (7.5) | 1.21 (1.16 - 1.26) | 1.81 (1.59 - 2.06) |
| Up to 15 | 63,070 | 3,223 | 69.6 (8.0) | 1.19 (1.15 - 1.23) | 1.70 (1.51 - 1.90) |
| Up to 20.6† | 63,070 | 3,289 | 70.2 (8.1) | 1.18 (1.14 - 1.22) | 1.66 (1.49 - 1.86) |

*age, 10 principal components of ancestry, sex, education level, smoking status, BMI, diabetes, hypertension, TC/HDL ratio, cholesterol lowering drugs.

†maximum follow-up

**Supplemental Table 3.** PCE Risk Groups in the High Genetic Risk Group (Quintile 5 of the PRS) by Statin Use and Race/Ethnic Groups in the GERA Cohort.

| **European** | | | | |
| --- | --- | --- | --- | --- |
|  | **Quintile 5 of the PRS** | | | **Full EUR Cohort** |
| **PCE Risk Group** | **Statin Use** | | **All** |  |
|  | **No** | **Yes** |  |  |
| Low (< 5%) | 3887 (58.4%) | 901 (25.7%) | 4788 (47.1%) | 23989 (46.3%) |
| Borderline (5 to < 7.5%) | 762 (11.4%) | 465 (13.3%) | 1227 (12.1%) | 6522 (12.6%) |
| Intermediate (7.5 to < 20%) | 1674 (25.1%) | 1483 (42.3%) | 3157 (31.0%) | 16127 (31.1%) |
| High (>= 20%) | 337 (5.1%) | 660 (18.8%) | 997 (9.8%) | 5201 (10.0%) |
| All | 6660 (100.0%) | 3509 (100.0%) | 10,169 (100.0%) | 51839 (100.0%) |

| **African-American** | | | | |
| --- | --- | --- | --- | --- |
|  | **Quintile 5 of the PRS** | | | **Full AA Cohort** |
|  | **Statin Use** | | **All** |  |
|  | **No** | **Yes** |  |  |
| Low (< 5%) | 51 (47.7%) | 6 (11.3%) | 57 (35.6%) | 705 (33.8%) |
| Borderline (5 to < 7.5%) | 11 (10.3%) | 6 (11.3%) | 17 (10.6%) | 281 (13.5%) |
| Intermediate (7.5 to < 20%) | 34 (31.8%) | 29 (54.7%) | 63 (39.4%) | 794 (38.1%) |
| High (>= 20%) | 11 (10.3%) | 12 (22.6%) | 23 (14.4%) | 304 (14.6%) |
| All | 107 (100.0%) | 53 (100.0%) | 160 (100.0%) | 2084 (100.0%) |
|  |  |  |  |  |
| **Latino** | | | | |
|  | **Quintile 5 of the PRS** | | | **Full LAT Cohort** |
|  | **Statin Use** | | **All** |  |
|  | **No** | **Yes** |  |  |
| Low (< 5%) | 263 (68.8%) | 64 (36.8%) | 327 (58.8%) | 2595 (59.7%) |
| Borderline (5 to < 7.5%) | 37 (9.7%) | 24 (13.8%) | 61 (11.0%) | 455 (10.5%) |
| Intermediate (7.5 to < 20%) | 70 (18.3%) | 57 (32.8%) | 127 (22.8%) | 928 (21.3%) |
| High (>= 20%) | 12 (3.1%) | 29 (16.7%) | 41 (7.4%) | 369 (8.5%) |
| All | 382 (100.0%) | 174 (100.0%) | 556 (100.0%) | 4347 (100.0%) |

| **Asian** | | | | |
| --- | --- | --- | --- | --- |
|  | **Quintile 5 of the PRS** | | | **Full AS Cohort** |
|  | **Statin Use** | | **All** |  |
|  | **No** | **Yes** |  |  |
| Low (< 5%) | 219 (73.0%) | 50 (31.6%) | 269 (58.7%) | 2924 (60.9%) |
| Borderline (5 to < 7.5%) | 22 (7.3%) | 22 (13.9%) | 44 (9.6%) | 484 (10.1%) |
| Intermediate (7.5 to < 20%) | 48 (16.0%) | 55 (34.8%) | 103 (22.5%) | 1039 (21.6%) |
| High (>= 20%) | 11 (3.7%) | 31 (19.6%) | 42 (9.2%) | 353 (7.4%) |
| All | 300 (100.0%) | 158 (100.0%) | 458 (100.0%) | 4800 (100.0%) |

**Supplemental Table 4.** Incremental utility and Reclassification among GERA Subjects by Subgroups.

| Statistic | Males  (n=20,569) | Females  (n=42,501) | European  (n=51,839) | African-American  (n=2,084) | Latino  (n=4,347) | Asian  (n=4,800) | All Minorities  (n=11,231) |
| --- | --- | --- | --- | --- | --- | --- | --- |
| AUC* Model1 | 0.688 | 0.737 | 0.745 | 0.719 | 0.762 | 0.755 | 0.752 |
| AUC* Model 2 | 0.695 | 0.741 | 0.749 | 0.720 | 0.765 | 0.757 | 0.755 |
| AUC* Difference | 0.107 | 0.004 | 0.004 | 0.001 | 0.003 | 0.002 | 0.003 |
| p-value | 0.007 | 0.002 | < 0.001 | 0.75 | 0.49 | 0.21 | 0.15 |
| IDI† All (95% CI) | 0.32 (0.15 - 0.55) | 0.21 (0.11 - 0.36) | 0.28 (0.16 - 0.44) | 0.08 (-0.01 - 0.86) | 0.33 (0.03 - 1.12) | 0.03 (-0.04 - 0.32) | 0.13 (0.01 - 0.38) |
| IDI† Intermediate Group (95% CI) | 0.37 (0.17 - 0.69) | 0.28 (0.13 - 0.54) | 0.33 (0.19 - 0.54) | 0.08 (-0.02 - 0.90) | 0.14 (-0.05 - 0.90) | 0.22 (0.00 - 0.86) | 0.14 (0.007 - 0.46) |
| NRI Categorical Entire Cohort (95% CI) | 2.9 (0.9 - 4.8) | 2.8 (0.5 - 5.0) | 2.6 (0.9 - 4.1) | -1.4 (-0.7 - 4.3) | -2.1 (-9.3 - 4.7) | 1.5 (-0.3 - 6.2) | 0.4 (-2.8 - 3.7) |
| NRI¶ Categorical Intermediate Group  (95% CI) | 9.5 (6.3 - 10.7) | 9.7 (6.3 - 13.8) | 9.8 (7.3 - 12.1) | 1.3 (-6.0 - 10.4) | 4.9 (-7.7 - 16.8) | 3.3 (-0.5 - 11.1) | 5.0 (-0.6 - 11.0) |
| NRI¶ Continuous Entire Cohort (95% CI) | 13.9 (8.9 - 18.6) | 18.7 (13.7 - 24.2) | 15.1 (11.3 - 19.0) | 15.3 (-5.6 - 36.7) | 18.8 (12.6 - 33.2) | 8.6 (-5.8 - 24.5) | 13.0 (3.1 – 22.9) |
| NRI¶ Continuous Intermediate Group  (95% CI) | 14.4 (7.9 - 21.1) | 19.1 (12.0 - 26.8) | 15.9 (0.10 - 21.1) | 11.8 (-14.0 - 39.0) | 17.7 (5.1 - 40.8) | 20.6 (-1.6 - 44.1) | 13.4 (0.4 – 27.4) |

*AUC: area under the curve; Model 1 is PCE risk alone; Model 2 is PCE risk + PRS

†: integrated discrimination improvement; ¶: net reclassification improvement

**
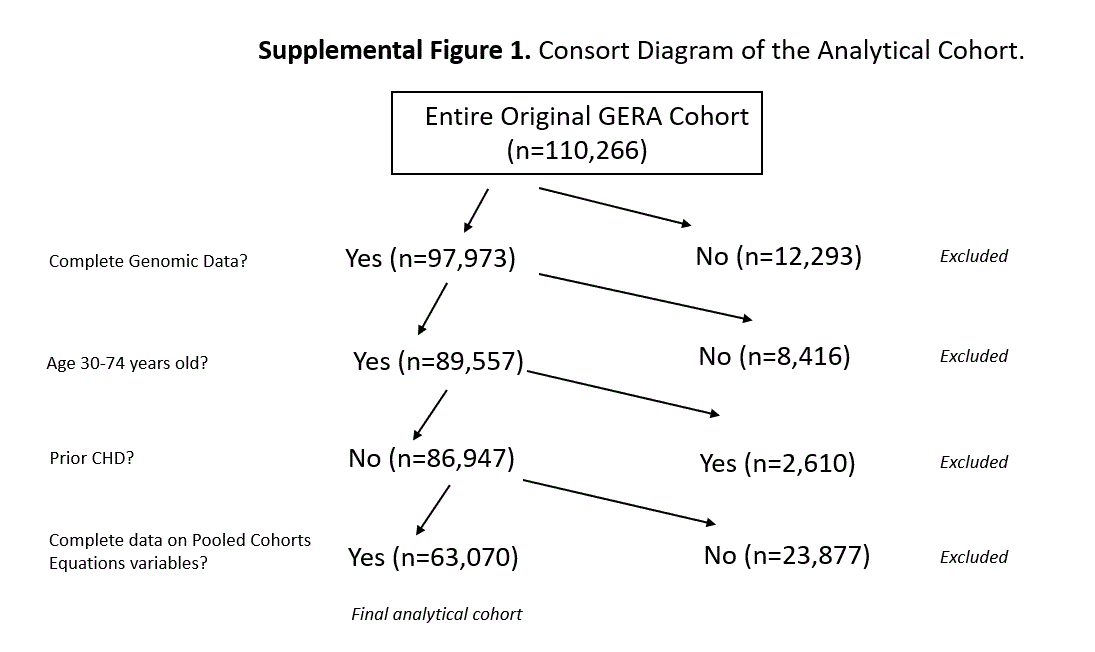
**

**Supplemental Figure 2.** Plot of Schoenfeldt residuals.


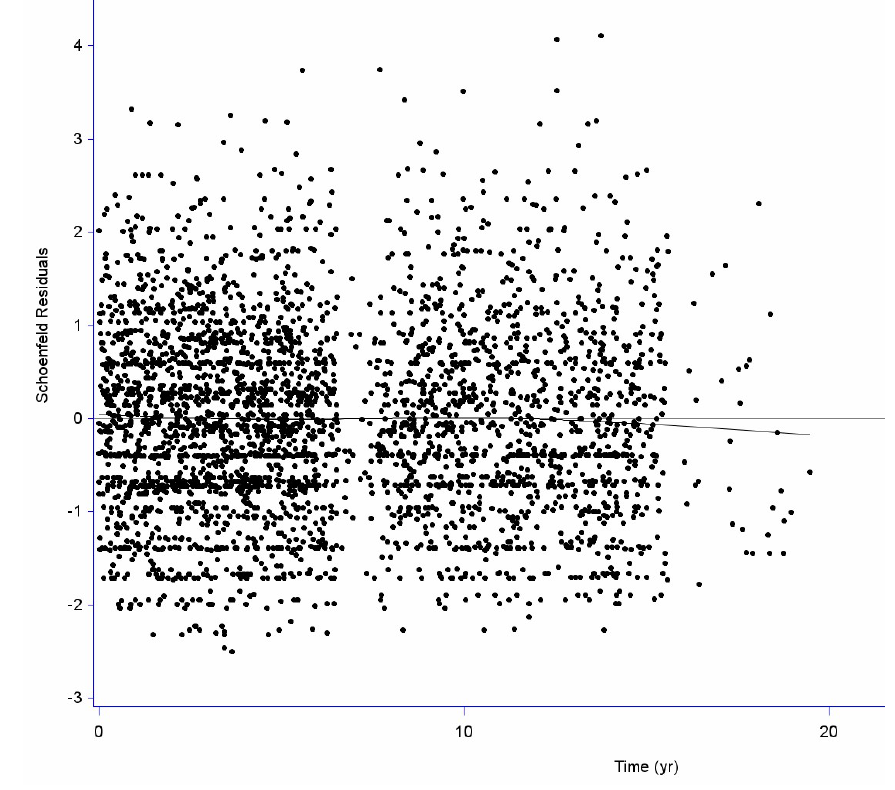


**Supplemental Figure 3.** Box Plots of PRS by ancestry groups.


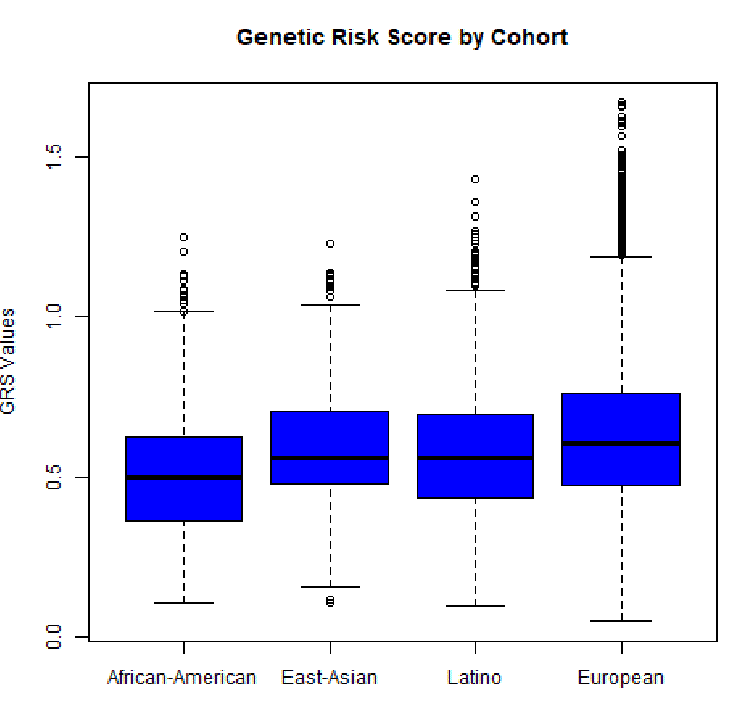


p-ANOVA<0.0001

PRS Value

African-American Asian Latino European

**Supplemental Figure 4.** Distribution of the PRS by incident CHD cases and non-cases (controls).


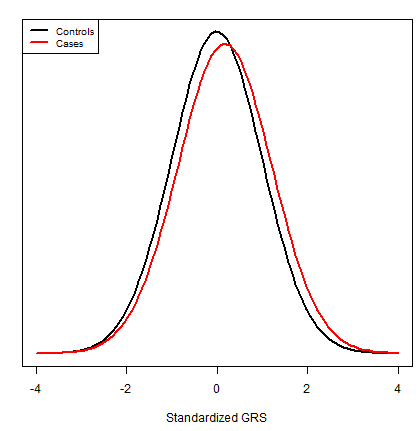


Mean difference = 0.04

(p<0.001)

**Standardized PRS**
